# Association between Disorders of Lipid Metabolism and Oculopathy: An Overview

**DOI:** 10.7150/ijms.116512

**Published:** 2025-08-22

**Authors:** Yuanting Yang, Ruixue Zhang, Miao Zhang, Zhaohui Yang, Zhongyu Ma, Yinqiao Zhang, Mengke Wu, Dadong Guo, Hongsheng Bi

**Affiliations:** 1 Shandong University of Traditional Chinese Medicine, Jinan 250002, China.; 2 Shandong Provincial Key Laboratory of Integrated Traditional Chinese and Western Medicine for Prevention and Therapy of Ocular Diseases; Shandong Academy of Eye Disease Prevention and Therapy, Jinan 250002, China.; 3 Medical College of Optometry and Ophthalmology, Shandong University of Traditional Chinese Medicine, Jinan 250002, China.; 4 Affiliated Eye Hospital of Shandong University of Traditional Chinese Medicine, Jinan 250002, China.

**Keywords:** metabolic disorder, eye diseases, cholesterol, lipoprotein, lipid deposition.

## Abstract

Lipid metabolism disorders, which lead to lipid deposition or changes in blood lipid composition, play a significant role in inducing or exacerbating the pathogenesis of diseases such as hypercholesterolemia and diabetes. Moreover, these disorders are closely associated with the development and progression of ocular diseases, including corneal degeneration, cataracts, glaucoma, age-related macular degeneration, and diabetic retinopathy. Studies have shown that lipid metabolism disorders critically impact the structure and function of ocular tissues through mechanisms such as lipid deposition, disrupted cholesterol synthesis, abnormal lipid concentrations, or impaired lipid transport. These disorders can damage cellular structures, induce oxidative stress, disrupt signal transduction, and lead to apoptosis of ocular tissue cells, mitochondrial dysfunction, and osmotic imbalance, ultimately impairing normal physiological functions and contributing to the onset of various eye diseases. This article reviews the association between lipid metabolism disorders and the development of various ocular diseases and explores the mechanisms underlying the interaction between lipid metabolism abnormalities and eye diseases, as well as the preventive role of lipid metabolism regulation in ocular diseases.

## 1. Introduction

Lipid metabolism is a complex process involving the synthesis, breakdown, transformation, and transport of lipid substances in organisms, essential for maintaining normal physiological functions and life activities. Lipids mainly comprise triglyceride (TG), cholesterol, and lipoproteins, which serve essential functions such as energy storage, cell membrane integrity, bioactive molecule synthesis/signaling, and lipid transport. Lipid metabolism disorders refer to pathological states characterized by abnormalities in the synthesis, degradation, or transport of lipids, resulting in dysregulated serum lipid levels (e.g., high cholesterol, high TG, low high-density lipoprotein cholesterol (HDL-C)) or metabolic dysfunction. These are collectively termed dyslipidemia, including abnormal lipid levels such as high/low cholesterol, hypertriglyceridemia, or imbalanced lipoprotein profiles (e.g., LDL/HDL). Throughout this review, unless otherwise specified, we use "dyslipidemia" to describe lipid metabolic disorders (e.g., hyperlipidemia, hypolipidemia.).

In recent years, with changing lifestyles and an aging population, the prevalence of lipid metabolism disorders and related diseases has been steadily increasing. Metabolic disorders such as hypercholesterolemia have become a major global health challenge 1. Analysis of national data from South Korea revealed that the prevalence of hypercholesterolemia rose from 9.0% in 2007 to 20.7% in 2018. In 2018, the overall prevalence of dyslipidemia was 45.6% in men and 31.3% in women, showing an age-dependent increase. The number of dyslipidemia cases surged nearly 8-fold, from 1.5 million in 2002 to 11.6 million in 2018 2. Similarly, dyslipidemia represents a significant public health burden in China. A study of Chinese adults found that 42.1% had dyslipidemia, low HDL-C as the most common subtype, followed by high TG (15.4%), high total cholesterol (TC) (8.3%), and high low-density lipoprotein cholesterol (LDL-C) (7.1%). Dyslipidemia was more common in men (47.3%) than women (38.8%) 3. Given these trends, exploring the association between lipid metabolism disorders and disease progression is crucial for improving global health outcomes.

Lipid metabolism plays a pivotal role in maintaining normal physiological functions. Cholesterol, serving as a precursor for steroid hormones, participates in the metabolism, stress response and reproductive functions. Abnormal cholesterol metabolism not only disrupts hormone secretion 4, but also interacts with glucose metabolism disorders to form "glucolipotoxicity", leading to mitochondrial dysfunction and reactive oxygen species production 5, thereby impairing normal physiological functions. Furthermore, lipoproteins transport lipids through the bloodstream to fulfill energy supply and tissue repair. Elevated lipoprotein levels can trigger inflammation, vascular stenosis/occlusion, consequently inducing atherosclerosis, cardiovascular diseases and diabetes 6, 7. This explains the growing body of research investigating the association between dyslipidemia and these related diseases. For instance: Atherosclerotic plaques are present in over 50% of hypercholesterolemia patients 8; the U.S. reported 613,969 deaths linked to coronary artery disease and dyslipidemia between 1999-2020 9; Approximately 96.6% of diabetic patients exhibit various patterns of dyslipidemia 10.

**Figure 1:**
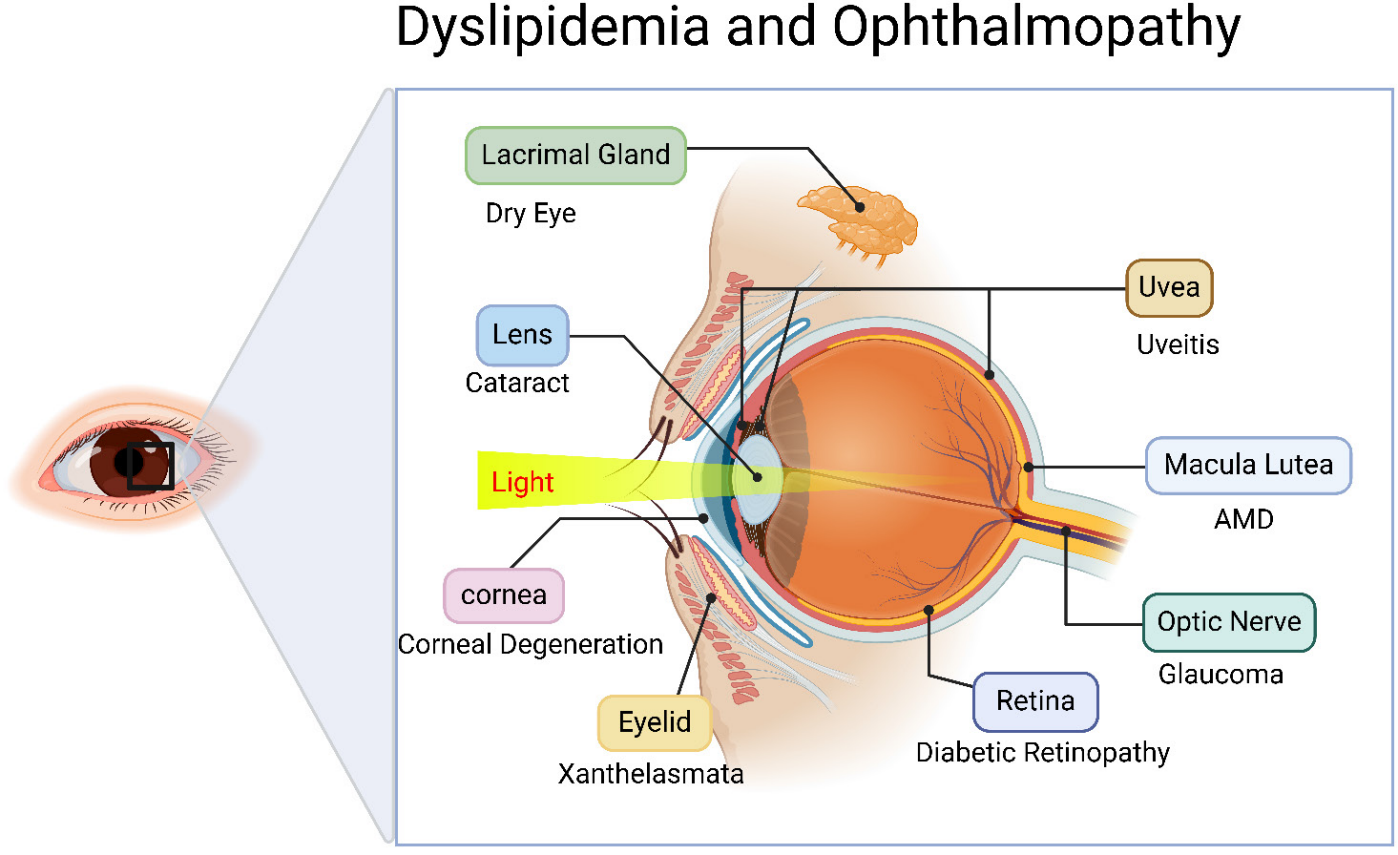
** Relationship between disorders of lipid metabolism and oculopathy.** Created in BioRender. 1, 1. (2025) https://BioRender.com/avh63pc.

However, lipid metabolism disorders leading to lipid deposition or functional impairment can adversely affect ocular health, potentially inducing sight-threatening ocular diseases impairing visual function. Consequently, it is critical to elucidate these associations and mechanisms. However, current research on dyslipidemia-associated major eye diseases (cataract, glaucoma, AMD, and DR) remains fragmented, lacking comprehensive systematic reviews. This study aims to critically review the mechanistic roles and research advances of lipid metabolism disorders in various ophthalmic pathologies, while exploring lipid modulation as a potential therapeutic strategy for ocular disease intervention (Figure 1).

## 2. Association Between Lipid Metabolism Disorders and Major Ophthalmic Diseases

### 2.1 Corneal diseases

#### 2.1.1 Corneal degeneration

Corneal degeneration refers to non-inflammatory, progressive structural deterioration and functional deterioration of corneal tissue caused by metabolic disorders, aging, diseases, or environmental factors. Among these conditions, corneal arcus (also known as arcus senilis) represents a characteristic lipid deposition disorder at the corneal periphery, with significantly higher prevalence in patients with familial hypercholesterolemia (FH) and hypertriglyceridemia. A study of 1,031 FH patients found corneal arcus in 36% of cases, with prevalence rising to ~45% by age 40 and increasing further with age 11. The Blue Mountains Eye Study, a cross-sectional investigation of 3,654 participants (82.4% aged ≥ 49 years), further established that corneal arcus was significantly associated with progressively elevated TC (> 5 mmol/L), hypercholesterolemia, and hypertriglyceridemia in elderly populations 12. The mechanistic links of corneal arcus in hypercholesterolemic patients may be mechanistically linked to: Single-base deletion (211delG) in the LDL receptor (LDLR) gene 13; Biallelic pathogenic variants (c.1069G > A and c.2034C > A) in LDLR 14; FH-related genetic mutations 15.

The first reported case analysis of dense deposit disease with corneal opacity and juvenile corneal arcus demonstrated a significant association between corneal manifestations and dyslipidemia, characterized by a markedly abnormal lipid profile including elevated serum TC (285 mg/dL; normal range: 112-232 mg/dL), elevated VLDL (66 mg/dL; normal range: 8.1-30.2 mg/dL), elevated low-density lipoprotein (LDL) (204 mg/dL; normal range: < 120 mg/dL), hypertriglyceridemia (334 mg/dL; normal range: < 150 mg/dL), and low high-density lipoprotein (HDL) (14 mg/dL; normal range: 27-77 mg/dL), with ocular dense deposits potentially linked to systemic lipid metabolism dysfunction 16. Studies of middle-aged Taiwanese populations further revealed that elevated TC, LDL-C, non-HDL-C, and TC/HDL-C ratios were significantly associated with increased corneal arcus risk 17.

Deficiency of lecithin-cholesterol acyltransferase (LCAT) or reduction in its activity or dysfunction may also lead to corneal arcus formation 18. This condition results from an LCAT defect caused by a single nucleotide substitution at codon 123 of the LCAT gene, which alters plasma lipoprotein composition and impairs cholesterol efflux capacity, particularly affecting HDL levels 19. Histopathological examination reveals extracellular vacuolization and amyloid deposition throughout stromal layers, with notable accumulation at Descemet's membrane 20. The underlying mechanism likely involves impaired cholesterol esterification, leading to deposition of unesterified cholesterol in the corneal stroma 21.

**Figure 2:**
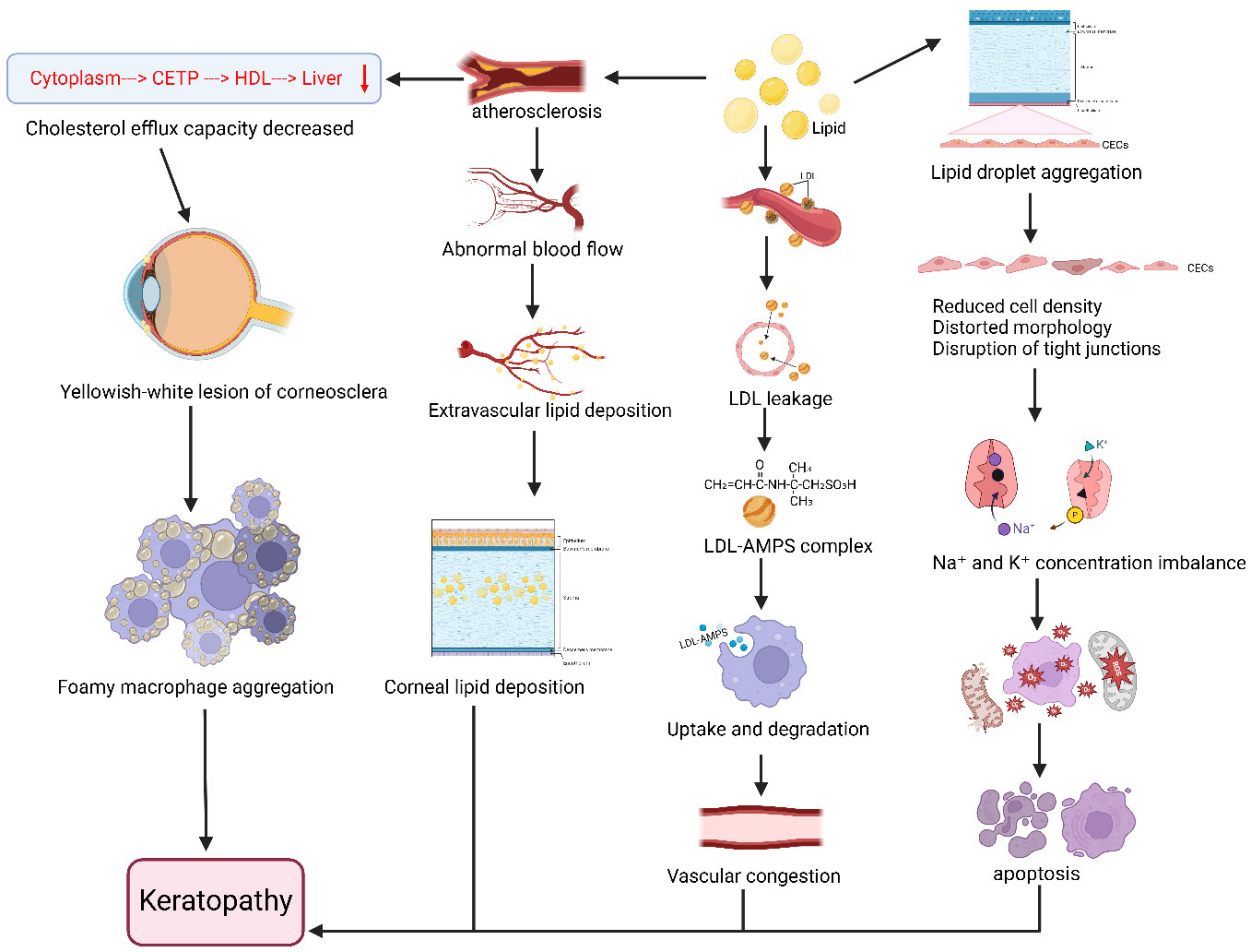
**Relationship between disorders of lipid metabolism and the progression of keratoconus.** Lipid accumulation in the body affects corneal health in a number of ways. Lipid deposition leads to the aggregation of lipid droplets within corneal endothelial cells (CECs), which results in decreased density of CECs, distorted cell morphology, disruption of intercellular tight junctions and adhesion junctions, reduction in the number of surface microvilli, reduction in the expression and function of intracellular Na+/K+-ATPase, imbalance in the concentration of sodium and potassium ions, activation of oxidative stress and mitochondrial dysfunction, and ultimately, corneal endothelial cytotoxicity and apoptosis. Meanwhile, lipids are initially distributed as low-density lipoproteins (LDL) around blood vessels and leak into tissues through an insulating process, after which LDL interacts with 2-acrylamide-2-methylpropanesulfonic acid (AMPS) to form LDL-AMPS complexes, which are selectively entrapped to different parts of the eye, and then phagocytosis uptakes and degrades the LDL-AMPS complexes and then releases the Cholesterol and other lipids are released into the ocular tissues, causing inflammatory congestion and vascular obstruction near the cornea. In addition to this, high lipid can lead to atherosclerosis, which on the one hand increases vascular permeability and abnormal blood flow, causing extravascular lipid deposition in ocular tissues, and ultimately lipid deposition occurs in the stromal layer of the cornea, forming corneal rings; on the other hand, the cycle of cytoplasm→cholesterol transfer protein (CETP)→high-density lipoprotein (HDL)→liver decreases, and the ability to exocytosis of cholesterol decreases, resulting in the occurrence of a corneo-scleral at yellowish-white lesions, foamy macrophage aggregates at the cornea, and corneal clouding. Together, these pathways contribute to the development and progression of corneal lesions. Created in BioRender. 1, 1. (2025) https://BioRender.com/rtkqkrf.

Animal studies have further confirmed that high-cholesterol diets can induce corneal lipid deposition 22, inducing intracellular lipid droplet accumulation in corneal endothelial cells (CECs). These dietary interventions cause disruption of tight junctions and adherens junctions in CECs, reduction of surface microvilli, downregulation of Na⁺-K⁺-ATPase expression and function, oxidative stress activation, mitochondrial ultrastructural alterations, and increased apoptosis. The pathological changes in corneal endothelium were found to correlate with dose-dependent cytotoxicity in CECs, morphological changes, decreased pump function, and oxidative stress induction 23. In rabbit models of hyper-ß-lipoproteinemia, ocular lipid deposition was observed primarily in the cornea, iris, and uveal tract. Lipids initially existed as LDL distributed perivascularly, later infiltrating tissues through insudation processes and becoming selectively entrapped in various ocular locations through interactions with acid mucopolysaccharides (AMPS). Phagocytic cells subsequently uptake and degrade LDL-AMPS complexes, potentially releasing cholesterol and other lipids into tissues and triggering inflammatory hyperemia, followed by sclerotic reactions. The study also revealed associations between hyperlipoproteinemia and increased vascular permeability, where additional congestion and enhanced permeability accelerated the rate and intensity of ocular lipid deposition 24. Furthermore, foam cell aggregation in the cornea may contribute to lipid accumulation 25. Muramatsu M et al. demonstrated that combined deficiency of apolipoprotein E (ApoE) and Down syndrome critical region-1 (Dscr-1) in Dscr-1^-/-^ and ApoE^-/-^ mice led to dramatic increases in serum cholesterol levels along with severe corneal opacity and complete penetrance 26. These symptoms were alleviated following treatment with atorvastatin (20 mg/d) and a low-cholesterol diet 27, demonstrating that lipid-lowering therapy can partially mitigate or prevent corneal degeneration (Figure 2).

Corneal degeneration arises from metabolic, age-related, and environmental factors, with dyslipidemia being a significant contributor to the development of corneal arcus. Notably, the juvenile corneal arcus may serve as an important clinical warning sign of underlying lipid metabolism disorders 28, 29. Lipid metabolism abnormalities thus play a pivotal role in the pathogenesis of corneal arcus, where genetic or protein-level disruptions in lipid pathways can induce structural changes in corneal tissue leading to arcus formation. Elucidating the mechanistic role of lipid metabolism in this pathological process and its associated tissue changes provides novel insights for developing targeted therapeutic strategies.

#### 2.1.2 Corneal dystrophy

Corneal dystrophies represent a group of progressive corneal disorders with familial inheritance patterns, among which lipid metabolism disorders play a critical role in the pathogenesis of Schnyder corneal dystrophy (SCD) 30 and Bietti crystalline dystrophy (BCD).

SCD is characterized by structural abnormalities of the anterior corneal stroma and Bowman's layer, featuring abnormal cholesterol and cholesterol esters deposits 31. Corneal crystalline deposits mimicking SCD's clinical phenotype 32 have been observed in hyperlipoproteinemia patients (particularly type IIa) 33, suggesting plasma lipoprotein abnormalities may induce localized metabolic disturbances in corneal tissue 34.

Studies of a French family 35 and a large Nova Scotian pedigree 36 revealed that this dystrophy originates from mutations in the UBIAD1 gene, which encodes prenyltransferase 1 - an enzyme involved in vitamin K1-to-K2 conversion and cholesterol biosynthesis. UBIAD1 mutations lead to dysfunctional prenyltransferase 1, resulting in systemic cholesterol crystal accumulation, corneal thickening, central corneal deposits, and potentially blindness in severe cases. Additional studies indicate SCD correlates with foam cell deficiency, lipid accumulation in corneal fibroblasts, and localized fibroblast apoptosis 37. BCD, caused by CYP4V2 gene mutations, results from defective lipid metabolism due to the encoded cytochrome P450 enzyme. Cyp4v3(-/-) mouse models demonstrate that CYP4V2 mutations induce systemic lipid metabolism disorders with subsequent lipid crystal deposition in both cornea and retina 38.

Therapeutic evidence suggests that low-cholesterol diets and intake of unsaturated fatty acids may improve corneal opacity and reduce cardiovascular risks 39. Studies of corneal stromal dystrophy (CSD) in Boxer dogs further support that low-fat diets improve lipoproteins and corneal lesions 40. These findings highlight the therapeutic potential of lipid metabolism regulation in corneal dystrophy, with lipid-lowering therapy a promising therapeutic strategy. Importantly, dietary modification and habit adjustment may prevent lipid metabolism disorder-induced corneal pathology.

### 2.2 Diseases of the ocular surface

#### 2.2.1 Dry eye

Dry eye disease (DED; keratoconjunctivitis sicca) is a chronic ocular surface disorder caused by abnormalities in tear quality/quantity or impaired tear film dynamics. Population studies in regions like Saudi Arabia revealed a 55.9% prevalence of dyslipidemia (95% CI 40.1-71.7) among DED patients, with significantly greater severity than the general population 41. A nationwide Korean survey of 17,364 adults (≥20 years) demonstrated that DED patients had higher systemic comorbidity rates, showing significant association between dyslipidemia (adjusted odds ratio: 1.63) and DED prevalence 42. Similar correlations were observed between dyslipidemia (elevated serum cholesterol and triglycerides) and DED in studies of 5,627 Korean women 43 and 15,294 general Korean adults 44. Notably, research on DED patients aged 25-70 years identified particular susceptibility in female populations 45. Retrospective analysis of 306 DED patients (18-87 years) further confirmed significantly increased DED incidence among women >40 years and dyslipidemic individuals 46. Systematic reviews and meta-analyses established that elevated TC, LDL-C, and HDL-C levels correlated with higher DED risk 47, especially in women 48. Cross-sectional studies also linked abnormal TC, HDL, LDL, and TG levels to DED progression, potentially through impaired tear film stability and meibomian gland dysfunction (MGD) 49.

Animal models provide mechanistic insights, showing that ACAT-1 deficiency with ApoE or LDLR knockout exacerbates dry eye symptoms by impairing lacrimal function and tear film stability 50. The underlying mechanism involves dysregulation of multiple oxidative stress pathways, characterized by significantly elevated key oxidative markers (4-HNE and MDA) and hyperactivation of oxidases (MPO, NOS3, and XOR). These alterations trigger inflammatory cascades through the TLR4/NF-κB pathway, while abnormally elevated systemic inflammation indices (NLR, PLR) reflect chronic inflammatory status. Notably, triglyceride accumulation in meibum directly activates these inflammatory pathways, establishing a vicious cycle between oxidative stress and inflammation 51, 52.

In conclusion, lipid metabolism may influence DED pathogenesis through lipoprotein-mediated pathways. Early DED screening for dyslipidemic patients could enable timely intervention to mitigate ocular health impacts. However, current evidence remains limited regarding precise mechanistic links between dyslipidemia and DED etiology, warranting further rigorous investigation into lipid-related pathophysiological roles. Furthermore, research on the application of lipid-lowering medications (particularly statins) in dry eye disease remains relatively scarce. Whether lipid-modulating therapies can prevent or alleviate dry eye symptoms requires further clinical investigation and validation.

#### 2.2.2 Meibomian gland dysfunction

The meibomian glands (MGs) primarily function to synthesize and secrete lipids that maintain tear film stability, prevent evaporation, and reduce friction. MGD is pathologically characterized by terminal duct obstruction and/or abnormal quality/quantity of meibum secretion. Dyslipidemia has been identified as a contributing factor to MGD development. A study of 116 patients aged 18-65 years demonstrated that MGD patients generally exhibited elevated serum lipid levels compared to healthy individuals 53, with particularly significant correlations observed between dyslipidemia 54 and MGD severity. Clinical observations of 66 moderate-to-severe MGD cases revealed a higher incidence of dyslipidemia compared to the general population, specifically showing elevated TC (> 200 mg/dL) in 67.4% vs. 45.1% of controls (P = 0.0012), while low HDL (HDL < 40 mg/dL) was observed in 6.5% vs. 15.7% of controls (P = 0.045) 55. Systematic reviews and meta-analyses 56 confirmed significant associations between dyslipidemia (particularly elevated TC and triglycerides) and MGD prevalence: among MGD patients, 20.0-77.6% had TC ≥200 mg/dL and 8.3-89.7% had TG ≥150 mg/dL, compared to 6.1-45.1% and 1.1-47.8% in age-matched controls.

Dyslipidemia may influence MG acinar cell differentiation, maturation, and lipid synthesis through both direct and indirect mechanisms 57. Excessive cholesterol accumulation alters meibum composition, disrupting the tear film lipid layer and aqueous retention, thereby exacerbating MGD pathology 58. Lipidomics studies and dyslipidemic mouse models have provided initial insights into the association between dyslipidemia and altered meibum composition 59. Research using obese mouse models demonstrated that high-fat diet-induced dyslipidemia resulted in meibomian gland hypertrophy, altered meibum composition, and increased saturated fatty acyl lipids in both plasma and meibum 60. Transcriptomic analysis revealed that dyslipidemia disrupts lipid metabolic homeostasis by disrupting rhythmic expression of core circadian clock genes (Clock, Per2, Per3) in meibomian glands, ultimately leading to gland dysfunction. These findings demonstrate a direct correlation between circadian rhythm disruption and morphological abnormalities of the glands, providing novel mechanistic insights into metabolism-associated meibomian gland dysfunction 61.

Notably, MG atrophy and deteriorated meibum quality may persist in dyslipidemic patients even during statin therapy 62. Therefore, the therapeutic efficacy of lipid-lowering therapy for meibomian gland dysfunction remains to be clinically validated.

In summary, dyslipidemia can contribute to MGD pathogenesis by altering meibum composition and impairing MG cellular function. However, current understanding of lipid metabolism's role in MGD pathogenesis remains limited. Future studies should further elucidate the specific role of dyslipidemia in MGD development to inform novel preventive and therapeutic strategies.

### 2.3 Eyelid diseases

#### 2.3.1 Xanthelasma

Xanthelasma is a lipid deposition disorder primarily caused by cholesterol accumulation in the eyelid region 63, representing a classic manifestation of lipid and lipoprotein metabolism disorders. Epidemiological investigations in Taiwan revealed that approximately 50% of xanthelasma patients have dyslipidemia 64, with hypercholesterolemia being particularly prevalent in this population 65. A study of 896 xanthelasma cases demonstrated uniform prevalence of hypercholesterolemia comorbidity across all age groups above 30 years 66. Cross-sectional studies further found an 80.00% dyslipidemia prevalence (95% CI 74-86) among xanthelasma patients 67. Clinical analyses indicate xanthelasma primarily affects middle-aged women, most commonly located on the upper eyelids 68. Nair PA's research validated this demographic trend, showing peak incidence in women aged 50-60 years, with associations to both dyslipidemia and potential early coronary artery disease 69.

The Lipid Research Clinics Population Study found highest prevalence xanthelasma prevalence in type II phenotype individuals, increasing with age. The condition is associated with elevated plasma cholesterol and LDL-C levels, particularly among young males 70. Notably, xanthelasma may develop even in normolipidemic patients, possibly due to atherogenic lipoprotein components 71. Chang HC's study demonstrated significantly increased apolipoprotein B levels (SMD, 1.036; 95% CI, 0.361 to 1.711; P = 0.003) and decreased atheroprotective apolipoprotein A1 (SMD, -0.328; 95% CI, -0.704 to 0.048; P = 0.087) in xanthelasma patients, potentially contributing to increased carotid intima-media thickness and elevated anti-atherogenic risk 72.

Therapeutic evidence shows cholesterol reduction reduces xanthelasma severity or induces regression xanthelasma. Probucol treatment in FH patients not only significantly reduced serum cholesterol but also induced xanthelasma regression 73, though non-macrophage lipid deposits may persist, suggesting probucol may redistribute cholesterol from macrophages to other cells. Furthermore, extremely low LDL cholesterol concentrations may lead to regression of cutaneous lipid disorders such as xanthelasma 74. Lipid-lowering therapy has also proven effective in treating familial hypercholesterolemia, thereby reducing xanthomatous manifestations including xanthelasma 75, 76. However, some patients may develop recurrence of lesions due to persistent lipid metabolism abnormalities despite treatment 77.

While existing studies confirm associations between dyslipidemia and xanthelasma, the precise mechanisms remain incompletely understood. Further research is needed to clarify the role of lipid metabolism in xanthelasma pathogenesis and develop targeted therapeutic strategies.

#### 2.3.2 Upper eyelid ptosis

Ptosis, as a common ocular condition, can impair visual acuity and function. Emerging evidence indicates associations between dyslipidemia and ptosis development. A study of 251 Japanese patients (aged ≥ 60 years) with age-related degenerative ptosis identified non-HDL cholesterol (p = 0.003) and HDL cholesterol (p = 0.044) as independent correlates, suggesting dyslipidemia as a potential risk factor for senile ptosis, though causal relationships require further investigation 78. Patients with mitochondrial DNA deletion-associated chronic progressive external ophthalmoplegia (CPEO) complicated by FH frequently exhibit bilateral ptosis alongside elevated serum cholesterol levels 79.

Certain genetic disorders demonstrate close links between impaired cholesterol biosynthesis and ptosis pathogenesis 80. For instance, Smith-Lemli-Opitz syndrome (SLOS), caused by DHCR7 gene mutations impairing cholesterol synthesis, leads to accumulated 7-dehydrocholesterol (7-DHC) disrupting normal cholesterol function in tissues, ultimately causing ptosis 81. Dhcr7(-/-) mouse models demonstrate that 7-DHC supports Ret signaling, but its deficiency impairs cholesterol synthesis, potentially compromising sympathetic nervous system function through disrupted Ret signaling pathways and inducing ptosis 82.

Lipid metabolism abnormalities may contribute to ptosis development by affecting neural transmission, tissue structure, and cellular signaling pathways. However, direct evidence linking dyslipidemia to ptosis remains limited, and Lipid-targeted therapies (e.g., cholesterol-lowering agents) are not yet established as primary interventions for blepharoptosis. Further in-depth investigations are required to elucidate the underlying pathological mechanisms and explore potential therapeutic approaches.

### 2.4 Cataract

Cataract, characterized by lens opacity due to reduced transparency or altered coloration of the crystalline lens, represents a degenerative change compromising optical quality. Substantial evidence establishes significant associations between dyslipidemia and cataract development. Analysis of 715,554 adults ≥ 40 years from the 2008-2012 Korean Community Health Survey demonstrated dyslipidemia as a significant risk factor for cataract 83. Park YH's study using 2008-2010 Korea National Health and Nutrition Examination Survey data demonstrated metabolic syndrome (MetS) and its components specifically correlated with age-related cataracts, particularly nuclear cataracts, in Korean women 84. Furthermore, Rim TH's research revealed a 40.1% cataract prevalence among adults > 40 years, with MetS and hypercholesterolemia identified as independent risk factors, suggesting cholesterol control may help reduce cataract incidence in Korea's general population 85. Cholesterol synthesis disorders and elevated cholesterol levels can induce lens fiber breakage, vacuolation 86, and retinal cell dysfunction 87, ultimately contributing to lens opacification.

Hypercholesterolemic rat models exhibit increased extracellular signal-regulated kinase (ERK) and malondialdehyde (MDA) alongside reduced glutathione (GSH), indicating enhanced oxidative stress 88. This oxidative imbalance further disrupts mitochondrial dynamics 89, leading to reactive oxygen species promote crystallin protein denaturation and aggregation—key processes in cataractogenesis. Cholesterol-induced mitochondrial oxidative stress also promotes intracellular lipoprotein accumulation 90. Spontaneously diabetic Torii fatty rats developed cortical and posterior subcapsular cataracts more rapidly than Sprague-Dawley controls, suggesting a synergistic effect by hypercholesterolemia and diabetes 91. High-fat diet mouse models demonstrated lipid peroxidation impairs Na^+^/K^+^-ATPase (NKA) function through NKAα1 subunit imbalance, disrupting oxidation-autophagy equilibrium and causing cellular damage 92. NKA dysfunction also alters ionic gradients, resulting in lens osmotic imbalance and cortical liquefaction.

**Figure 3:**
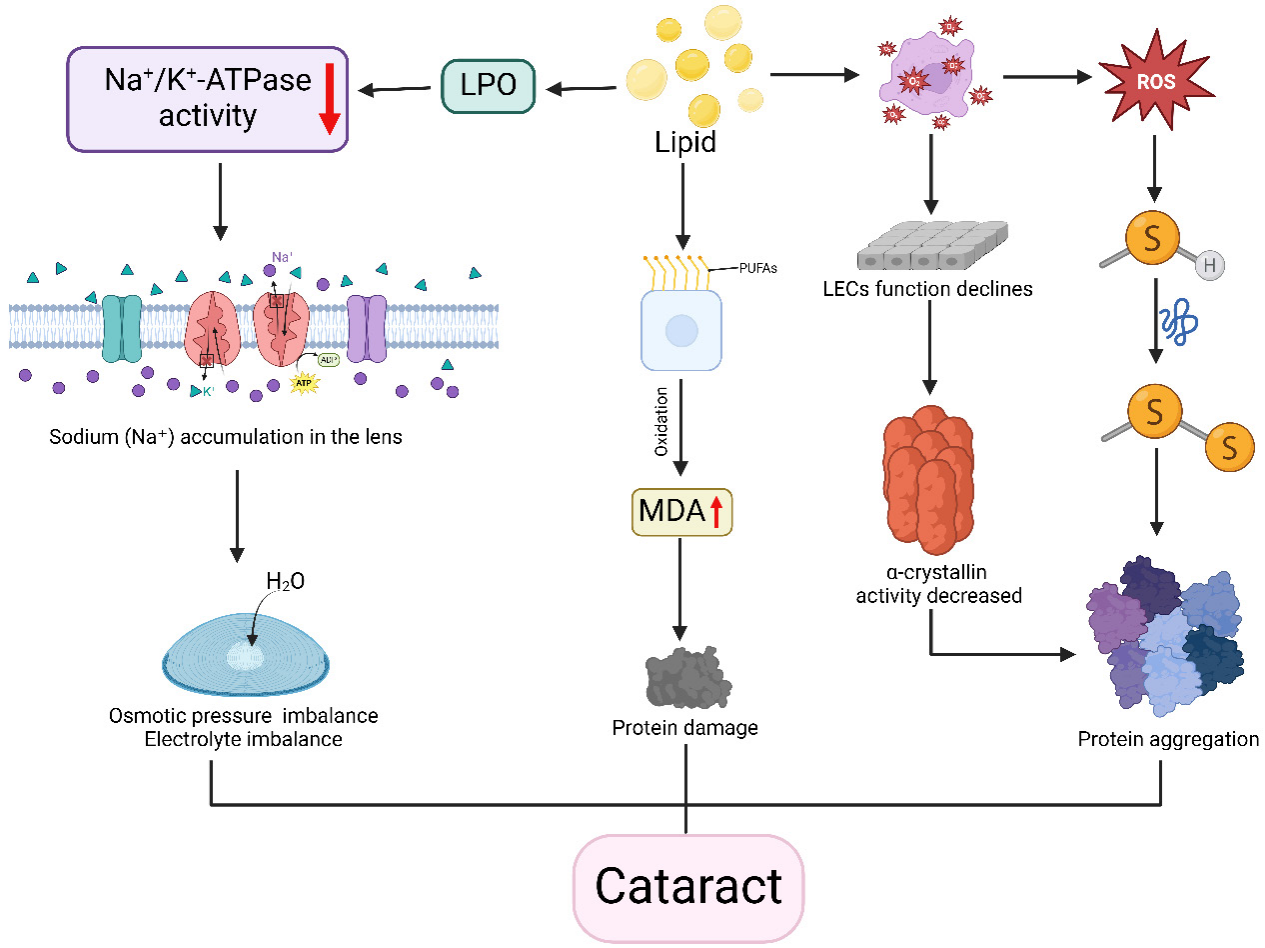
**Association between disorders of lipid metabolism and cataracts.** Disorders of lipid metabolism and imbalances in lipid peroxide (LPO) levels reduce the activity of the Na+/K+-ATPase enzyme, leading to sodium ion accumulation in the lens, osmotic pressure and electrolyte imbalances, and water influx into the lens, resulting in lens edema and cortical liquefaction. Also, metabolic disorders cause oxidation of polyunsaturated fatty acids PUFAs in cell membranes to produce malondialdehyde (MDA), resulting in protein damage. In addition, lipid imbalance also triggers oxidative stress. On the one hand, the generated reactive oxygen species attack protein sulfhydryl groups (-SH), denaturing proteins and forming disulfide bonds (-S-S-), which leads to protein aggregation; on the other hand, oxidative stress decreases the function of lens epithelial cells and reduces the function of α-lens proteins, which can likewise lead to denaturation and aggregation of proteins. The combined effect of these mechanisms contributes to the development of cataract. Created in BioRender. 1, 1. (2025) https://BioRender.com/rtb0ufa.

Genetic disorders of cholesterol metabolism like SLOS and cerebrotendinous xanthomatosis (CTX) also are associated with cataracts 93. SLOS involves post-squalene pathway defects leading to elevated specific sterol intermediates that induce cataracts 94, while CTX - caused by CYP27A1 mutations impairing mitochondrial sterol 27-hydroxylase - leads to cholesterol accumulation resulting in cataracts and tendon xanthomas 95. A Chinese case report of cerebrotendinous xanthomatosis patients showed that lipid-lowering therapy normalized serum free fatty acid levels and alleviated disease-related symptoms 96 (Figure 3).

Collective evidence indicates lipid metabolism dysregulation as a significant cataract risk factor, particularly through cholesterol-related pathways. Maintaining lipid homeostasis may help prevent or delay cataract onset, though targeted interventions based on lipid abnormalities warrant further exploration.

### 2.5 Glaucoma

Glaucoma, a disease characterized by optic nerve damage due to elevated intraocular pressure (IOP), has been increasingly linked to dyslipidemia in its pathogenesis 97. A study of 18,161 Italian glaucoma patients identified hypertension (60.2%) as the most prevalent comorbidity, followed by dyslipidemia (29.7%) 98. Baseline data from the China Health and Retirement Longitudinal Study demonstrated significant association between glaucoma and dyslipidemia (OR 1.757; 95% CI 1.157-3.650), with notable regional prevalence variations 99. Analysis of 2015-2021 Korea National Health and Nutrition Examination Survey data confirmed dyslipidemia (OR 1.529) as a glaucoma risk factor 100. Case-control studies revealed abnormal TG levels increased primary open-angle glaucoma (POAG) risk by 16.9-fold compared to normolipidemic individuals 101. Younger-onset POAG showed stronger dyslipidemia association (OR 1.49; 95% CI 1.07-2.08) versus elderly patients 102. Mendelian randomization supports dyslipidemia as an independent glaucoma predictor 103, where HDL-C inversely correlates with risk 104 while LDL-C 105 and TG show positive associations 106. Dysregulated LDL metabolism alters trabecular meshwork cholesterol homeostasis, modulating mechanical signaling and aqueous humor outflow 107. Lipid deposits in the trabecular meshwork induce chronic inflammation, fibrosis, and extracellular matrix remodeling, ultimately elevating IOP. GPR18 lipid receptor signaling activation similarly modulates murine IOP.

**Figure 4:**
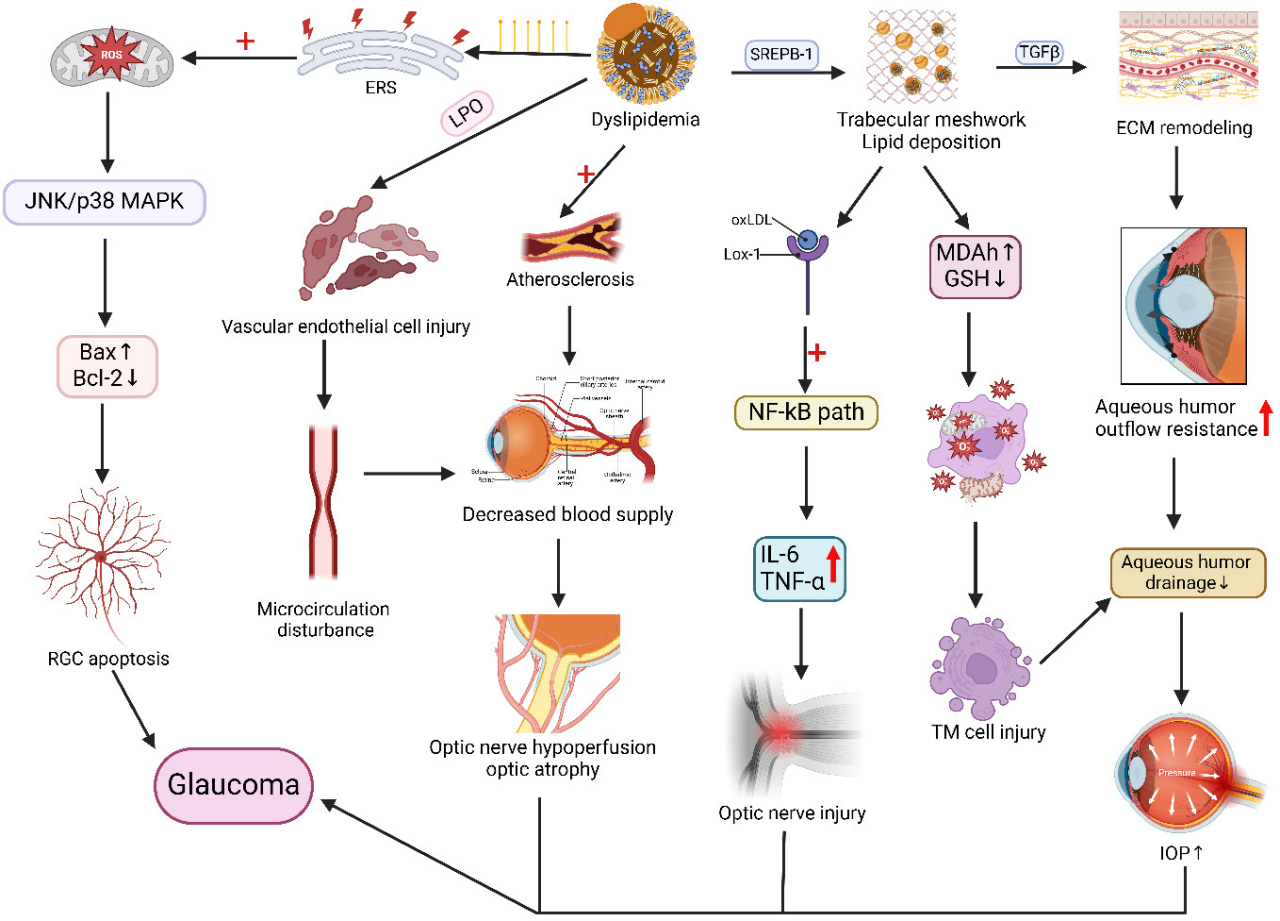
**Link between disorders of lipid metabolism and the pathogenesis of glaucoma.** Lipid metabolism disorders, with increased saturated fatty acids in the body, trigger endoplasmic reticulum stress (ERS), exacerbate mitochondrial dysfunction and reactive oxygen species production, activate the JNK/p38 MAPK pathway, and an imbalance in the Bax/Bcl-2 ratio, causing apoptosis of the retinal ganglion cells (RGC). Lipids also promote atherosclerosis and vascular endothelial cell damage, resulting in microcirculatory impairment of blood flow, reduced blood supply, and decreased blood supply to the optic nerve from the short posterior ciliary artery, leading to optic nerve atrophy. In addition, lipid disorders affect SREPB-1 expression, resulting in lipid deposition in the trabecular meshwork, causing lectin-like oxidized LDL receptor-1 (Lox-1) uptake of oxidized LDL, activation of the NF-kB signaling pathway, and elevated expression of IL-6 and TNF-α, resulting in inflammatory damage to the optic nerve. Lipid deposition in the trabecular meshwork also causes oxidative stress and remodeling of the trabecular meshwork extracellular matrix (ECM), resulting in trabecular meshwork cellular damage, increased resistance to outflow of aqueous humor, and impaired aqueous drainage, which ultimately leads to elevated intraocular pressure. The mechanisms of action, such as changes in intraocular pressure and optic nerve atrophy, are coordinated to promote glaucoma in the figure. Created in BioRender. 1, 1. (2025) https://BioRender.com/qwzyxcw.

Hypercholesterolemia may exacerbate glaucoma through NOS-2-mediated oxidative damage to retinal ganglion cells 108 or vascular dysfunction impairing optic nerve perfusion 109, 110. Comparative studies demonstrate elevated oxidative status, stress indices, and TC in normal-tension and pseudoexfoliation glaucoma patients versus controls, with altered PON1 enzyme activity 111, suggesting oxidative stress and dyslipidemia contribute across glaucoma subtypes (Figure 4).

Statins demonstrate protective effects against glaucoma progression, particularly in patients exhibiting intraocular pressure (IOP) reduction but persistent visual field loss. These agents may reduce glaucoma risk and serve as a novel therapeutic strategy 112, 113. Talwar N et al. further demonstrated that compared to non-users, patients receiving continuous statin therapy for 2 years exhibited a 21% reduction in glaucoma risk (adjusted HR, 0.79; 95% CI, 0.66-0.96; P = 0.02) 114. In JAMA Ophthalmology, Kang et al. 115 analyzed three large longitudinal cohorts and established an inverse association between prolonged statin use (≥5 years) and primary open-angle glaucoma (POAG) incidence. In addition, LDL-C lowering medications demonstrate efficacy in reducing glaucoma risk, providing additional therapeutic rationale for dyslipidemic glaucoma patients 116.

Collectively, dyslipidemia is significantly associated with glaucoma pathogenesis. Emerging evidence suggests anti-glaucoma medications may exert therapeutic effects through systemic lipid profile modulation, potentially regulating intraocular pressure via pathogenic lipid reduction.

### 2.6 Retinopathy

The retina, a critical component of the human visual system, is essential for receiving and transmitting visual information. Dyslipidemia has been strongly associated with the pathogenesis and progression of various retinal diseases, with studies showing that abnormal lipid profiles increase the risk of DR 117, 118, retinal vein/artery occlusion (RVO/RAO) 119, age-related macular degeneration (AMD), and retinal microvascular abnormalities 120, among others.

#### 2.6.1 Diabetic retinopathy

In DR, Shi R et al. found that dyslipidemia significantly correlates with reduced retinal blood flow in patients, potentially leading to thinning of the retinal nerve fiber and ganglion cell layers 121. Abnormal lipid metabolism is strongly associated with both the onset and severity of DR 122, with apolipoproteins such as apoB and apoC-II/apoC-III are implicated in DR pathogenesis while apoA-I/A-II may serve protective roles 123. Strict control of serum apolipoprotein B (apoB) levels may serve as an effective therapeutic strategy for DR.

**Figure 5:**
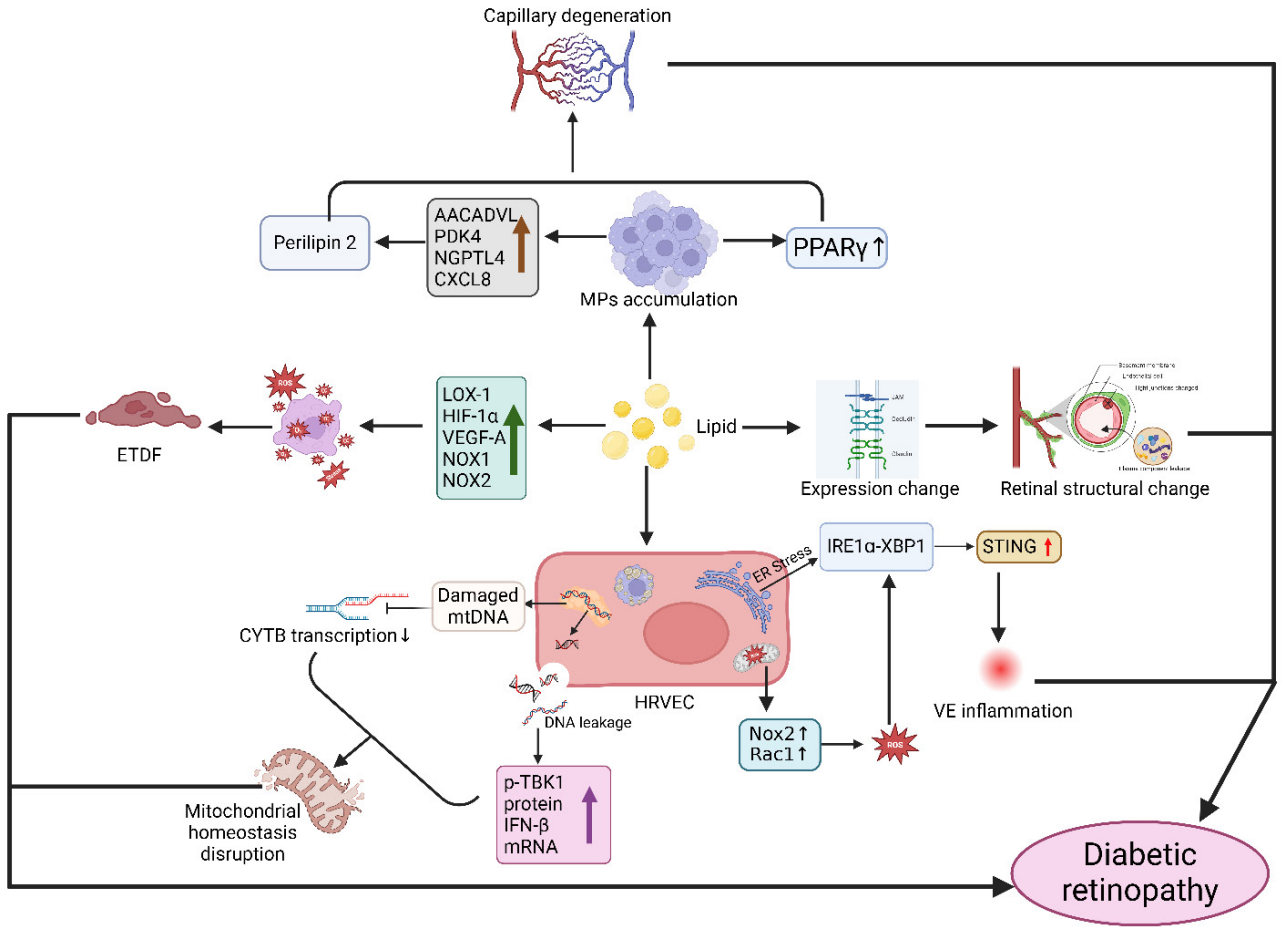
**Mechanism of action between disorders of lipid metabolism and the development of diabetic retinal degeneration.** Disorders of lipid metabolism cause aggregation of the monocyte macrophage system (MPS), which activates PPARγ signaling and affects Perilipin2 protein expression, resulting in capillary degeneration. Lipid deposition also causes changes in the expression of intercellular tight junction proteins (JAM, Occludin, and Claudin), which increases the permeability of the blood-retinal barrier and the infiltration of plasma components into the retina, causing changes in retinal structure. Abnormal lipid metabolism also affects the expression of various factors (LOX-1, HIF-1α, VEGF-A, NOX-1, NOX-2), activating oxidative stress and causing endothelial dysfunction (ETDF). In addition, lipid abnormalities lead to endoplasmic reticulum stress and mitochondrial dysfunction, reactive oxygen species (ROS) generation, and activation of STING signaling through the IRE1α-XBP1 axis in human retinal endothelial cells (HRVECs), triggering vascular endothelial (VE) inflammatory responses. Meanwhile, mitochondrial DNA from retinal endothelial cells leaks into the cytosol, and glycolipotoxicity damages mtDNA and affects cytochrome B (CYTB) transcription, ultimately leading to disruption of mitochondrial homeostasis. Created in BioRender. 1, 1. (2025) https://BioRender.com/ncg8p6q.

During DR progression, increased extravasation of modified LDL is observed, with highly oxidized glycated LDL (HOG-LDL) exacerbating DR progression by inducing caspase activation, mitochondrial dysfunction, and pericyte apoptosis in human retinal capillary pericytes 124. Additionally, lipotoxicity can induce accumulation of mononuclear phagocytes expressing the lipid-load marker Perilipin 2 in retinal regions, where pro-inflammatory macrophages further promote capillary degenerative changes 125. Dyslipidemia may also disrupt the blood-retinal barrier, leading to extravasation and local modification of lipoproteins that ausing significant tissue damage 126. Studies in Zucker diabetic fatty (ZDF) rats, a type 2 diabetes model, demonstrated that dyslipidemia induces staged retinal alterations: elevated cytoplasmic reactive oxygen species (ROS) at early stages (6 weeks), mitochondrial dysfunction at intermediate phases (12 weeks), and mitochondrial DNA damage in advanced disease (≥20 weeks), indicating lipid metabolism disorders accelerate retinopathy through oxidative stress and mitochondrial impairment by disrupting energy metabolism and induce apoptosis 127. In type 1 diabetic mouse models, extravasated modified plasma lipoproteins drive DR progression, with intravitreal injection of human HOG-LDL causing severe progressive retinal damage characterized by morphological abnormalities, ERG changes, vascular leakage, VEGF overexpression, gliosis, endoplasmic reticulum stress, and apoptosis 128 (Figure 5).

For diabetes-related complications, lipid-lowering therapy offers a potential therapeutic approach. The 2024 AMD Annals Program revealed that 72.0% of patients showed improvement with lipid-modifying medications 129. This therapeutic feasibility was further corroborated by Italian diabetic patients attaining LDL target levels through lipid-lowering regimens 130.

#### 2.6.2 Age-related macular degeneration

Age-related macular degeneration (AMD), a chronic progressive degenerative disease of the macular region, primarily involves pathological changes in the retinal pigment epithelium (RPE)-Bruch's membrane-choriocapillaris complex. The Blue Mountains Eye Study in Australia demonstrated that metabolic syndrome components including obesity, hyperglycemia, and hypertriglyceridemia correlate with increased AMD risk during 10-year follow-up of participants aged ≥49 years 131, 132.

**Figure 6:**
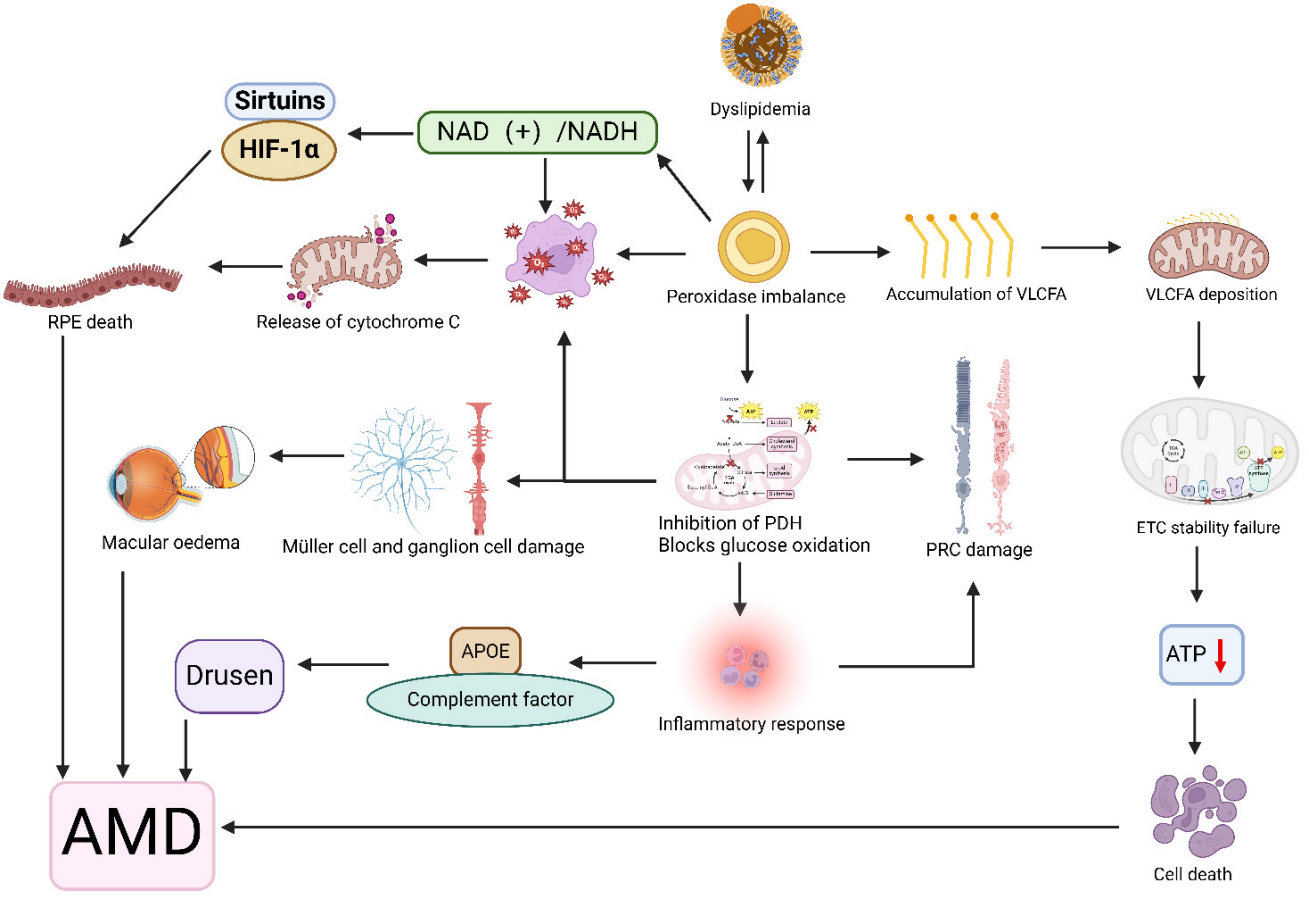
**Illustration of the association between disorders of lipid metabolism and age-related macular degeneration (AMD).** Lipid metabolism disorders affect peroxidase expression levels, and the interaction between the two is such that peroxidase accumulates very long chain fatty acids (VLCFA), which are deposited on mitochondrial membranes affecting membrane fluidity, destabilizing the electron transport chain (ETC) complex, and leading to reduced ATP production and cell death. Peroxidase also blocks glucose oxidation by inhibiting pyruvate dehydrogenase (PDH) activity, leading to retinal photoreceptor cell (PRC), muller cell, and ganglion damage, macular edema, and an inflammatory response that results in an imbalance of apolipoproteins (APOEs) and complement, leading to the development of vitreous warts. In addition peroxidase induces oxidative stress and changes in the NAD(+)/NADH redox state, leading to the release of cytochrome C from the mitochondria, dysregulation of the expression of Sirtuins and HIF-1α, and ultimately retinal pigment epithelial (RPE) cell death. Together, these mechanisms contribute to the development of AMD. Created in BioRender. 1, 1. (2025) https://BioRender.com/exw8si8.

Dyslipidemia may impair retinal function by disrupting intracellular organelle homeostasis. Peroxisomes, which are critical for lipid metabolism (including fatty acid oxidation, phospholipid synthesis, and ROS regulation), become dysregulated under lipid overload. This leads to oxidative stress, mitochondrial lipid deposition, and inflammatory damage, contributing to drusen formation, macular injury, and RPE/photoreceptor apoptosis 133. Abnormal apolipoprotein E metabolism also triggers retinal inflammation and drusen generation 134. While lipid accumulation damages RPE cells, it concurrently injures Bruch's membrane 135, 136, ultimately causing photoreceptor degeneration and retinal dystrophy 137. Animal models of Cyp27a1(-/-)Cyp46a1(-/-) mice confirm that cholesteryl ester accumulation may accelerate photoreceptor damage and macular degeneration 138 (Figure 6).

Furthermore, multiple studies have demonstrated that lipid-lowering medication use exerts potentially beneficial effects on AMD prevalence, reducing AMD risk by 35-50% 139, 140. These findings provide additional therapeutic potential for managing age-related macular degeneration.

#### 2.6.3 Retinal occlusive diseases

In retinal occlusive diseases, studies have identified dyslipidemia as a potential key factor, with 5 out of 6 patients (83.3%) with concurrent branch retinal artery and vein occlusion showing abnormal lipid profiles. This suggests lipid metabolism disorders may contribute to these rare vascular events through atherosclerosis, increased blood viscosity, and endothelial dysfunction 141. A large multicenter Italian case-control study identified dyslipidemia (OR: 2.255 [1.352-3.762], p=0.002) as an independent risk factor for central retinal vein occlusion (CRVO) 142. Multiple studies demonstrate hypercholesterolemia, hypertriglyceridemia, and low HDL-C are independently associated with retinal vein/artery occlusion (RVO/RAO) 143-145, possibly through mechanisms such as increased plasma viscosity, endothelial dysfunction, or thrombogenesis 146, 147. Porcine models of hypercholesterolemia show retinal ultrastructural changes mediated by increased oxidative stress, NO metabolites, and superoxide anion release 148.

#### 2.6.4 Other retinopathies

Lipid abnormalities are implicated in other retinopathies like lipemia retinalis, where severe hypertriglyceridemia induces milky retinal vasculature which resolves with dietary intervention 149. Murine models indicate dyslipidemia modifies circRNA expression profiles, regulating focal adhesion signaling and endothelial function during retinal vascular dysfunction 150. Additionally, dyslipidemia induces retinal microvascular damage 151, manifesting as arteriolar narrowing, reduced vessel density, and nerve fiber layer thinning, mediated by endothelial dysfunction and inflammation 152.

In summary, the key pathological mechanisms include: oxidative stress and inflammation via Nox2-derived ROS activation of STING/IRE1α-XBP1 pathways 153, 154; vascular dysfunction driven by cholesterol-induced endothelial activation and impaired microcirculation 155; and metabolic-autophagy imbalance where lipid accumulation disrupts energy homeostasis and exacerbates neurodegeneration 156, 157. Statins and fenofibrate may mitigate DR progression through combined lipid-lowering, anti-inflammatory, and antioxidant effects to reduce macular edema and vascular leakage 158, 159. Optimized dyslipidemia management (e.g., statins, fibrates) improves retinopathy outcomes 160, highlighting its therapeutic relevance. Lipid metabolism disturbances impact retinal structure/function through multifaceted mechanisms. Future research should elucidate molecular links between lipid profiles and retinal microstructural changes to inform novel treatment strategies.

### 2.7 Myopia

Myopia, a common refractive error, occurs when parallel light rays focus anterior to the retina when the eye is in a relaxed state, resulting in blurred distance vision while maintaining relatively clear near vision. Emerging evidence links myopia development to lipid metabolism. Che et al. identified five differential lipids in myopic patients, with three—BMP (20:3/22:3), PS (14:1/22:4), and TG (55:3)_FA18:1—showing significant correlations with spherical equivalent refraction. BMP and PS additionally associated with axial length 161. SWATH-based quantitative proteomics revealed marked lipid metabolic alterations in early-stage myopic guinea pig retinas 162. Mendelian randomization demonstrated causal relationships between myopia and intermediate-density lipoprotein components: TC (OR 0.90, 95% CI 0.86-0.95, P=0.00021) and cholesteryl esters (OR 0.85, 95% CI 0.79-0.92, P=0.0006) 163.

Myopic patients exhibit significant retinal lipid peroxidation 164, which may further induce damage to both retinal tissue and ganglion cells 165. *In vitro* studies suggest human scleral fibroblasts' bioactivity promotes scleral elongation through sterol regulatory element-binding protein-mediated regulation of fatty acid, TG, and cholesterol synthesis - a proposed key mechanism for axial elongation 166. Murine myopia models show elevated LILRB2/Pirb protein expression promotes fatty acid synthesis and lipid accumulation, disrupting choroidal function and accelerating pathological myopia progression 167. High-fat diet-fed mice demonstrate excessive retinal lipid droplet accumulation, elevated oxidative stress and inflammatory signaling, accompanied by RPE degeneration, Bruch's membrane thickening, and photoreceptor dysfunction 168. Studies in dragon-eye goldfish reveal PPAR signaling pathway upregulation concurrent with increased lipid accumulation may trigger ocular morphological changes resembling myopic defects 169.

While myopia pathogenesis involves multifactorial mechanisms, lipid metabolism abnormalities contribute through disrupted lipid synthesis, peroxidation, and retinal structural alterations. However, current research has not yet identified lipid-lowering therapy as an effective treatment for myopia, and whether reducing relevant lipid levels can prevent or slow myopia progression remains unverified. Further research should elucidate precise roles of lipids and metabolites in myopia development to inform preventive strategies against pathological myopia and its complications.

## 3. Conclusion

In summary, lipid metabolism is closely associated with ocular health, maintaining visual function, retinal homeostasis, and cellular membrane stability. Lipids serve not only as essential structural components of ocular tissues including the retina, cornea, optic nerve, and aqueous humor but also participate in critical biological processes such as signal transduction, energy metabolism, and immune regulation. Dyslipidemia, beyond its systemic metabolic effects, is implicated in various ocular pathologies including xanthelasma, glaucoma, corneal disorders, and DR. These diseases likely arise from imbalances in ocular lipid composition, deficiency of key enzymes, or genetic mutations, which induce pathological changes such as lipid deposition, inflammatory responses, oxidative stress, osmotic imbalance, and cellular apoptosis, ultimately leading to structural and functional impairments of ocular tissues.

Despite established associations between dyslipidemia and ocular diseases, several challenges remain. The precise dynamics of lipid metabolic cycles within ocular tissues and their underlying molecular mechanisms require further elucidation. Although lipid abnormalities can be mitigated through dietary modifications, pharmacological interventions, and lifestyle adjustments, the pathogenesis of ophthalmic diseases involves complex interactions among multiple factors. Future research should investigate the interplay between lipid metabolism and other contributing elements (e.g., genetic predisposition, inflammation, vascular abnormalities) to develop more effective prevention and treatment strategies.

## Abbreviations

AMD: age-related macular degeneration
LDL: low-density lipoprotein
HDL: high-density lipoprotein
FH: familial hypercholesterolemia
LDLR: low-density lipoprotein receptor
LDL-C: low-density lipoprotein cholesterol
LCAT: lecithin-cholesterol acyltransferase
SCD: Schnyder corneal dystrophy
BCD: Bietti crystalline dystrophy
DED: Dry eye disease
SLOS: Smith-Lemli-Opitz syndrome
TC: total cholesterol
TG: triglyceride
MGD: Meibomian gland dysfunction
7-DHC: 7-dehydrocholestero
HDL-C: high-density lipoprotein cholesterol
IOP: intraocular pressure
DR: diabetic retinopathy
RPE: retinal pigment epithelium
